# LncRNA00638 promotes the osteogenic differentiation of periodontal mesenchymal stem cells from periodontitis patients under static mechanical strain

**DOI:** 10.1186/s13287-023-03404-6

**Published:** 2023-07-11

**Authors:** Xiaochen Zhang, Qing Yan, Xulin Liu, Jie Gao, Yuerong Xu, Zuolin Jin, Wen Qin

**Affiliations:** 1 State Key Laboratory of Military Stomatology and National Clinical Research Center for Oral Diseases and Shaanxi Clinical Research Center for Oral Diseases, Department of Orthodontics, School of Stomatology, Fourth Military Medical University, Xi’an, 710032 China; 2 State Key Laboratory of Military Stomatology and National Clinical Research Center for Oral Diseases and Shaanxi Key Laboratory of Stomatology, Department of Prosthodontics, School of Stomatology, Fourth Military Medical University, Xi’an, 710032 China

**Keywords:** LncRNA00638, Periodontal mesenchymal stem cells, Osteogenic differentiation, Orthodontic tooth movement, Periodontitis

## Abstract

**Background:**

The osteogenic differentiation capacity of periodontal mesenchymal stem cells (PDLSCs) can be influenced by different levels of static mechanical strain (SMS) in an inflammatory microenvironment. Long non-coding RNAs (lncRNAs) are involved in various physiological processes. However, the mechanisms by which lncRNAs regulate the osteogenic differentiation of PDLSCs remain unclear.

**Methods:**

We investigated the responses of PDLSCs obtained from periodontitis patients and healthy people to 8% and 12%SMS. Gene microarray and bioinformatics analyses were implemented and identified lncRNA00638 as a target gene for the osteogenesis of PDLSCs from periodontitis patients under SMS. Competing endogenous RNA (ceRNA) network analysis was applied and predicted interactions among lncRNA00638, miRNA-424-5p, and fibroblast growth factor receptor 1 (FGFR1). Gene expression levels were regulated by lentiviral vectors. Cell Counting Kit-8 assays, alkaline phosphatase assays, and Alizarin Red S staining were used to examine the osteogenic potential. RT-qPCR and Western blot were performed to detect the expression levels of related genes and proteins.

**Results:**

We found that 8% and 12% SMS exerted distinct effects on HPDLSCs and PPDLSCs, with 12% SMS having the most significant effect. By microarray analysis, we detected differentially expressed lncRNAs/mRNAs between 12% SMS strained and static PPDLSCs, among which lncRNA00638 was detected as a positive target gene to promote the osteogenic differentiation of PPDLSCs under SMS loading. Mechanistically, lncRNA00638 may act as a ceRNA for miR-424-5p to compete with FGFR1. In this process, lncRNA00638 and miR-424-5p suppress each other and form a network to regulate FGFR1.

**Conclusions:**

Our findings demonstrate that the lncRNA00638/miRNA-424-5p/FGFR1 regulatory network is actively involved in the regulation of PDLSC osteogenic differentiation from periodontitis patients under SMS loading, which may provide evidence for optimizing orthodontic treatments in patients with periodontitis.

**Supplementary Information:**

The online version contains supplementary material available at 10.1186/s13287-023-03404-6.

## Background

Periodontitis is an inflammatory infectious disease of tooth-supporting tissues with a high incidence rate that produces aesthetic and functional problems for patients [1]. Orthodontic treatment is the optimal choice for patients with periodontitis to establish the occlusion and restore masticatory function, which are beneficial to periodontal health. In this process, periodontal mesenchymal stem cells (PDLSCs) play an essential role in bone remoulding due to orthodontic tooth movement. However, increasing evidence has shown that the decreased osteogenic ability of PDLSCs in the inflammatory microenvironment may be the cause of intolerance to orthodontic forces in patients with periodontal diseases [2, 3]. Static mechanical strain (SMS) is often used to simulate the stress under which PDLSCs are subjected during OTM in vitro. Our research group found that the osteogenic differentiation ability of PDLSCs can be influenced by different levels of SMS [4]. Exploring the causes and related mechanisms of the different responses of PDLSCs to different levels of SMS can provide a theoretical basis for the rational clinical application of orthodontic forces.

Long non-coding RNAs (lncRNAs) which are more than 200 nt in length and do not encode proteins, are involved in autoimmune, cardiovascular, and neurodegenerative diseases at the posttranscriptional level [5]. In recent years, the biological roles of lncRNAs in osteogenic differentiation and immune inflammation have been confirmed by many researchers [6]. Some lncRNAs, such as POIR, ANCR and XIST, can regulate the osteogenic differentiation of PDLSCs [7–10], but the underlying molecular mechanisms are still poorly understood. LncRNAs can act as different functional molecules, including molecular signals, decoys, guides and scaffolds, in gene regulation processes [11]. Among these functions, lncRNAs have been proposed to act as competing endogenous RNAs (ceRNAs) in a newly proposed model of gene regulation [12–14]. In this novel hypothesis, lncRNAs act as ceRNAs to sponge microRNAs(miRNAs), thereby inhibiting miRNAs from regulating their target genes [15–17]. For example, the lncRNA-POIR has been shown to promote the osteogenic differentiation of PDLSCs by competing with the osteogenesis-related target gene FoxO1 for binding miR-182 and blocking the inhibitory effect of miR-182 on FoxO1 [10]. However, whether lncRNAs regulate the osteogenic differentiation process of PDLSCs obtained from the periodontitis patients (PPDLSCs) under SMS is yet to be elucidated.

In this study, we identified a key gene, lncRNA00638, through gene microarray and bioinformatics analysis, and found that lncRNA00638 may positively regulate the osteogenic differentiation of PPDLSCs under mechanically loaded conditions in the periodontitis microenvironment. During this process, lncRNA00638 and miR-424-5p mutually repress one another to regulate fibroblast growth factor receptor 1 (FGFR1), the target gene of miR-424-5p, which affects the osteogenic differentiation of PPDLSCs. This study has identified a new target for improving the osteogenic capability of PPDLSCs under SMS and providing optimized orthodontic treatment in periodontitis patients.

## Methods

### Sample selection and cell cultures

HPDLSC tooth samples were obtained from 11 volunteers with an average age of 31.9 ± 5.2 years who did not have periodontal diseases. PPDLSC tooth samples were taken from 9 patients with a mean age of 36.1 ± 6.2 years who were diagnosed with moderate chronic periodontitis. The patients with periodontitis all had periodontal probing depth (PD) ≤ 6 mm, an attachment loss (AL) of 3–4 mm, and horizontal resorption of the alveolar bone of up to 1/3–1/2 of the root length. None of the selected volunteers had any systemic diseases, or a history of smoking, radiation therapy or chemotherapy. All procedures were approved by the Ethical Committee of the School of Stomatology, Fourth Military Medical University (IRB-REV-2020005). All participants provided written informed consent.

The periodontal tissues were scraped from the middle 1/3 of the root from the tooth samples. Collagenase type I (Gibco/Invitrogen, Carlsbad, CA, USA) was added to digest the periodontal tissues in a CO_2_ incubator at 37 °C for 1 h. The digestion was terminated by the addition of an equal volume of α-minimum essential medium (α-MEM, Gibco/Invitrogen, Carlsbad, CA, USA) containing 10% (v/v) foetal bovine serum (FBS), 1% (v/v) penicillin, and 1% (v/v) streptomycin. The obtained cells were inoculated in 6-well plates until they reached 80% confluence and isolated by the limiting dilution technique for subsequent experiments. All subsequent experiments were performed with 3rd-generation cells.

### SMS loading

Third-generation cells were inoculated in BioFlex 6-well plates at a density of 2 × 10^6^ cells/well and incubated at 37 °C for 12 h. Cells were synchronized by the addition of serum-free α-MEM medium for 24 h of starvation, and then placed on the FX-4000T Draft Force Loading System (Flexcell, Burlington, USA) for SMS loading. SMS with a force value of 8% or 12% at a frequency of 0.1 Hz were applied to HPDLSCs and PPDLSCs for 12 h. The morphology of the cells before and after SMS loading was observed by inverted microscopy (Olympus, Tokyo, Japan).

### Cell viability assays

Cell viability was analysed by Cell Counting Kit-8 (CCK-8, Kanglang Biological Technology Co. Ltd., Shanghai, China) assays. After SMS loading, cells were inoculated into 96-well plates, and 6 replicate wells and 1 blank control well were set for each group. Ten microlitres of CCK-8 solution was added to each well and incubated at 37 °C for 4 h. Then, an enzyme linked immunosorbent assay (ELISA, Biotek, Vermont, USA) was used to detect the absorbance (OD) at 490 nm. The analysis was repeated three times to obtain the statistical data.

### Gene microarray and data analysis

Gene microarray was used to analyse differentially expressed lncRNAs and mRNAs before and after 12% SMS loading. PPDLSC samples were divided into the control group and the 12% SMS group. Total RNA was extracted using TRIzol reagent, and the quantity and quality of the RNA were measured with a NanoDrop ND-1000 (Biosystems, USA), followed by RNA purification. The mRNA was extracted from the total RNA, and the mRNA from ach sample was amplified by the random start method and transcribed into fluorescent cRNA along the entire transcriptome length. The labelled cRNA was purified using an RNeasy Mini Kit (Invitrogen, Carlsbad, CA, USA), and the concentration and activity of the labelled cRNA were measured with a Nano Drop ND-1000. After the lncRNA expression microarrays were assembled, they were incubated in an Agilent hybridization oven (Agilent, Santa Clara, CA, USA) at 65 °C for 17 h. The hybridized arrays were washed, fixed and scanned using an Agilent DNA microarray scanner (Axon GenePix 4000B, Axon, USA). The obtained array images were analysed using Agilent Feature Extraction software v11.0.1.1 (Agilent, USA). Raw data were processed using the Gene Spring GX v11.5.1 software package (Agilent, USA), and lncRNAs and mRNAs from at least 2 of the 4 samples with current or marginal “all target values” were selected for further data analysis. Differentially expressed lncRNAs and mRNAs between the two groups were identified by volcano map filtering. Image data analysis was performed using Nimble Scan software (Agilent, USA), and hierarchical clustering was performed using Agilent GeneSpring GX software v11.5.1 (Agilent, USA).

### Bioinformatics analysis

We searched the Kyoto Encyclopedia of Genes and Genomes (KEGG) database to determine the biochemical pathways enriched in the differentially expressed mRNAs, Gene Ontology (GO) analysis examines the enrichment of differentially expressed mRNAs in three domains to investigate their potential functions: biological process (BP), cellular component (CC), and molecular function (MF). Through coding-noncoding gene co-expression (CNC) analysis, we found mRNAs whose expression patterns were the same as those of specific lncRNA and predicted the biological functions. Among the studied samples, we screened out several lncRNAs with a fold change > 50.0, *P* < 0.05 and raw intensity > 100. Then, RT- qPCR analysis of these selected genes was performed to confirm the microarray results and to select the most significantly differentially expressed lncRNA as the target gene. To explore the mechanistic pattern of the target gene, lncRNA00638, we searched miRbase (http://www.mirbase.org/), TargetScan *(*http://www.targetscan.org/mamm/*)*, and UCSC (http://genome.ucsc.edu/) for information on lncRNA–microRNA interactions. Additionally, ceRNA analysis was carried out to identify potential targeted microRNAs based on TargetScan and MiRanda. By merging the targeted microRNAs, we constructed a ceRNA network. The ceRNA analysis was completed by Kangcheng Biological Engineering (Shanghai, China).

### Lentiviral transfection

PPDLSCs were transfected with lentiviral vector to regulate the expression of lncRNA00638. The lentiviral vector was designed and constructed by GeneChem Co. Ltd. (Shanghai, China). Third generation PPDLSCs were seeded on 96-well plates at a density of 1 × 10^4^ cells/ml. Complete medium and ENi.S were added to dilute polybrene respectively and the final concentration was set at 5 µg/ml. When the cell confluence rate approached 1/3, transfection was carried out according to the determined viral titre. The infection efficiency was detected by fluorescence microscopy. mRNA was extracted for RT-qPCR to confirm the expression of lncRNA00638. PPDLSCs were divided into 5 groups after lentiviral transfection and SMS loading. For the transfection of anti-miR424-5p, PPDLSCs were seeded on BioFlex 6-well plates at a density of 1 × 10^5^ cells/ml. Cells were synchronized 24 h before transfection in serum-free medium. Transfection was carried out when the cell confluence rate reached 2/3. RT-qPCR was used to confirm the expression of anti-miR424-5p.

### Alizarin red S (ARS) staining and quantification

ARS staining and quantification were used to qualitatively and quantitatively detect osteogenic differentiation ability respectively. After SMS loading, the cell density was adjusted to 1 × 10^5^ cells/well in 6-well plates. When the cells had proliferated to reach 80% confluence, the original culture medium was replaced with osteogenic induction solution (α-MEM with 10% FBS, 10^−8^ mol/l dexamethasone, 10 mmol/l β-glycerophosphate sodium, 50 µg/ml vitamin C). After 21 days, the cells were rinsed 3 times with PBS, fixed with 4% paraformaldehyde for 30 min, and then rinsed repeatedly with distilled water. ARS solution (Sigma, Shanghai, China) was added to each well and incubated at 37 °C for 20 min. Then the staining solution was discarded, and the cells were rinsed with double-distilled water for 3 times. Images were captured with an inverted microscopy. Additionally, 500 µL/per well cetylpyridinium chloride was added to the plates for quantitative detection and incubated at 37 °C for 30 min, after which 150 µL of the mixture was added to a 96-well plate, and the OD value at 520 nm was measured by ELISA.

### Alkaline phosphatase (ALP) assay

Cells were seeded in 96-well plates at a density of 2 × 10^3^ cells/well and repeatedly rinsed with double-distilled water for 3 times, fixed with 4% paraformaldehyde at 37 °C for 45 min, and rinsed again with double distilled water. The cells were stained according to the instructions of an ALP kit (Beyotime, Shanghai, China) and incubated for 45 min in a constant-temperature incubator after addition of the staining solution. Then, the staining solution was removed, and the cells were rinsed with double-distilled water. Images were captured by inverted microscopy, and the OD value at 520 nm was measured by ELISA.

### Real-time quantitative polymerase chain reaction (RT-qPCR)

HPDLSCs and PPDLSCs were collected, and total RNA was isolated with TRIzol (Invitrogen, Carlsbad, CA, USA). The total RNA was converted to complementary DNA (cDNA) with a Prime Script™ RT-qPCR reverse transcription kit (Takara, Shiga, Japan). cDNA) was used as a template for the following steps. The SYBR Green method was performed to amplify target fragments. Primer sequences for osteogenesis-related genes and lncRNAs are listed in Table 1.

**Table 1:** Primer sequences for osteogenesis-related genes and lncRNAs

Gene	Forward primer (5′–3′)	Reverse primer (5′–3′)
β-actin	TGGCACCCAGCACAATGA	CTAAGTCATAGTCCGCCTAGAAGCA
Runx2	CCCGTGGCCTTCAAGGT	CGTTACCCGCCATGACAGTA
ALP	GGACCATTCCCACGTCTTCAC	CCTTGTAGCCAGGCCCATTG
OCN	CCCAGGCGCTACCTGTATCAA	GGTCAGCCAACTCGTCACAGTC
ENST00000550947	AAGCTTGGCTGTGTCTGTCTAGTG	GCGGATGCCTTCTCTCTAGTCTT
uc011jsq.2	TAATTGAGAAGAGGCAGGAAATCAC	TTCAAGGTACAATCCTCCTTCTGG
ENST00000456176	AGCCAGACCTAGGCAGTATTTCAG	CCACAACAATTGAAACTGCAGTATG
LncRNA00638	GACCAGTGGAACTGATGGACACA	CTTGGGATTCCACAAATGACACT
ENST00000562852	CCTTAGAAGAAGAGGAGATTGGG	GGCAAGGTTGGTTTCATTCTG
AK021458	GCTCTGGGTACCAAGGACACA	CCACCCTTTCTGGTCATTTGC
TCONS_00014557	GTTGAGGGTGCAGTGAGTTGTG	AATGGGTGCTCTTGGAGAGTTT
TCONS_00008000	ACCTGTGAAGATGGAAGGAACC	TTCAAGGTACAATCCTCCTTCTGG

### Western blotting

Radioimmunoprecipitation assay (RIPA) (Bio-Rad, USA) lysis buffer was used to extract the total proteins. The total protein concentration was quantified by the bicinchoninic acid protein assay method (Bio-Rad, USA) according to the manufacturer’s protocol. Protein samples were dissociated by sodium dodecyl sulfate–polyacrylamide (SDS–PAGE) gel electrophoresis and transferred to a PVDF membranes (Millipore, USA). The membranes were washed with blocking solution (TBST) for 45 min and then incubated with antibodies. The target signals were detected by enhanced chemiluminescence (ECL) detection (Peiqing Technology Co., Ltd, Shanghai, China).

### Statistical analysis

IBM SPSS statistical package 26.0 (IBM Co., Chicago, USA) was used for statistical analysis. Categorical variables are presented as the mean ± standard deviation (SD). Two independent samples *t* test was selected for comparisons between two independent groups. One-way ANOVA with Student–Newman–Keuls (SNK) comparison was employed for multiple comparisons among multiple different groups. Pearson’s test was used for correlation analysis. A significance value of 0.05 was chosen.

## Results

### PPDLSCs and HPDLSCs responded differently to SMS, with 12% SMS having the most significant force value

**Fig. 1:**
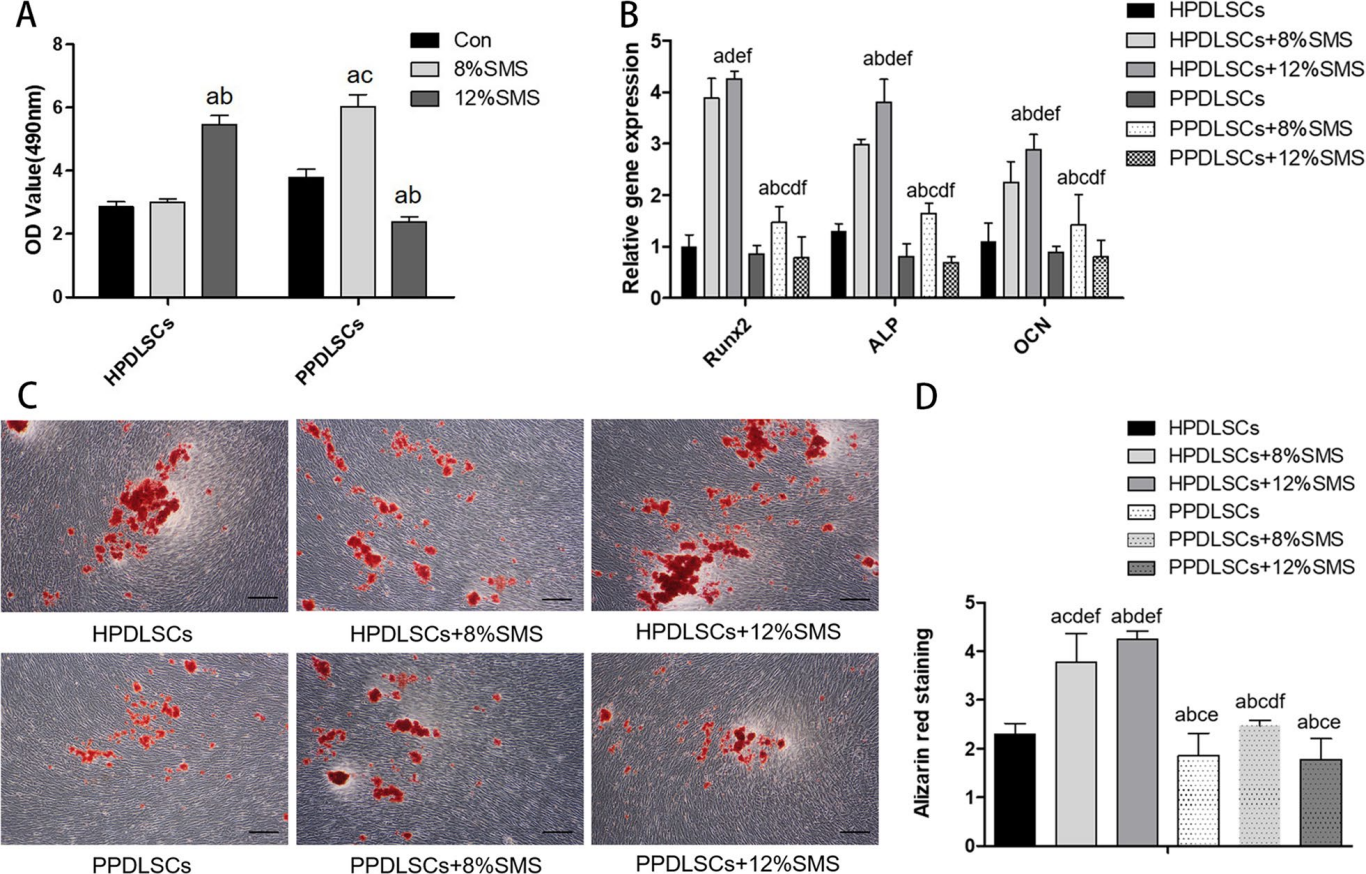
Cellular responses to different levels of SMS. **A** CCK-8 assay was used to analyse the proliferation ability of HPDLSCs and PPDLSCs under 12% and 8% SMS. **B** RT-PCR was used to detect the expression levels of osteogenesis related genes. **C** ARS staining was used to detect osteogenic differentiation. Scale bar = 100 µm. **D** ARS quantitative assay. All experiments were repeated three times. All data are shown as the mean ± SD. a: compared with the HPDLSCs group (*P* < 0.05); b: compared with the HPDLSCs + 8%SMS group (*P* < 0.05); c: compared with the HPDLSCs + 12%SMS group (*P* < 0.05); d: compared with the PPDLSCs group (*P* < 0.05); e: compared with the PPDLSCs + 8%SMS group (*P* < 0.05); f: compared with the PDLSCs + 12%SMS group (*P* < 0.05)

The proliferative ability of HPDLSCs and PPDLSCs under 12% and 8% SMS were examined by CCK-8 assay. Measurement of the OD value in each group demonstrated that the quantity of HPDLSCs under 12% SMS was significantly higher than that of the 8% SMS group and the control group, whereas compared to that of HPDLSCs, the quantity of PPDSCs was higher under 8% SMS but lower under 12% SMS (Fig. 1A). The osteogenic differentiation ability was detected by RT-qPCR analysis of osteogenic genes and ARS staining. The results showed the following: (1) Under 12% SMS, the relative expression levels of the osteogenic genes Runx2, ALP, and OCN were increased in HPDLSCs compared with the control group, but there was no significant difference in PPDLSCs. After osteogenic induction for 21 days, mineralized nodules in the HPDLSCs + 12%SMS group were more abundant in quantity and more condensed in distribution than those in the control group, but there was no significant difference in the number of mineralized nodules between the PPDLSCs + 12%SMS group and the control group. (2) Under 8% SMS, the expression of osteogenic genes in HPDLSCs was also increased compared with that in the control group but was less than that in the 12% SMS group. Interestingly, in PPDLSCs, the levels of osteogenic genes were significantly higher in the 8% SMS group than in either the control or 12%SMS groups. The ARS staining results were essentially consistent with the RT-qPCR data, that is, mineralized nodules in HPDLSCs + 8% SMS group were more densely distributed than those in the control group but less dense than those in the 12% SMS group, while that the mineralized nodules in the PPDLSCs + 8% SMS group were more densely distributed than those in the control group or the 12% SMS group (Fig. 1B, C). The quantitative ARS assay results were consistent with the changes revealed by RT-qPCR (Fig. 1D). These results suggest that PPDLSCs and HPDLSCs responded differently to SMS. Under 12% SMS, they showed the most significant differences in osteogenic differentiation and proliferation capacities.

### Expression profiles of lncRNAs and mRNAs under SMS

**Fig. 2:**
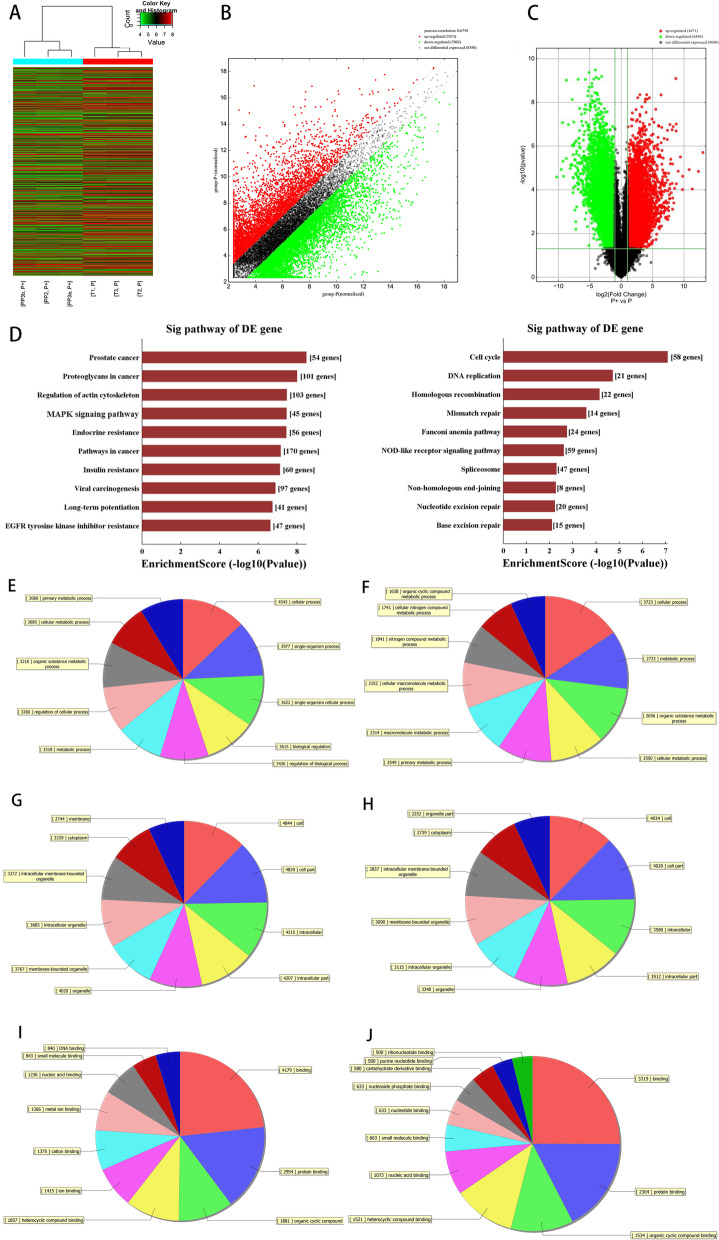
Microarray screening and analysis. **A** Hierarchical clustering and heatmap analysis were used to detect differentially expressed lncRNAs after 12% SMS loading. T1, T2, and T3 are control group samples taken from PPDLSCs without SMS loading, and PP1, PP2, and PP3 are experimental group samples taken from PPDLSCs with SMS loading. There were visible differences between the two groups. **B** Scatter plot showing variations in the expression of lncRNAs. Black dots are lncRNAs with a differential expression ratio < 2.0, red and green dots are lncRNAs with a differential expression ratio > 2.0. **C** Volcano plot showing the relationship between the fold changes in the expression of differentially expressed lncRNAs among sample groups and the corresponding statistical significance. Black dots indicate *P* > 0.05 and red and green dots indicate *P* < 0.05. **D** KEGG pathways enriched in mRNAs with increased expression (left) and decreased expression (right). **E** Top 10 BPs enriched in mRNAs with increased expression. **F** Top 10 BPs enriched in mRNAs with decreased expression. **G** Top 10 CCs enriched in mRNAs with increased expression. **H** Top 10 CCs enriched in mRNAs with decreased expression. **I** Top 10 MFs enriched in mRNAs with increased expression. **J** Top 10 MFs enriched in mRNAs with decreased expression

Gene microarray was used to identify the differential lncRNA expression profiles of PPDLSCs after 12% SMS loading. Heatmap (Fig. 2A), scatter plots (Fig. 2B), and volcano plot analyses (Fig. 2C) showed that 4471 lncRNAs and 7077 mRNAs were upregulated; and 6444 lncRNAs and 5816 mRNAs were downregulated after 12% SMS loading. To further explore the putative functions of these lncRNAs and mRNAs, bioinformatic analysis based on KEGG pathway and GO analyses was applied. The results showed that the actin cytoskeletal pathway and MAPK pathway which are mainly involved in cellular biomechanical signalling and osteogenic differentiation regulation, scored high in the KEGG analysis (Fig. 2D). Among the enriched GO terms, “single-multicellular organism process” and “mitotic cell cycle process” among BPs, “cell junction” and “nuclear part” among CCs, and “protein binding” and “RNA binding” among MFs earned the highest enrichment scores. We found that the items identified by the GO analysis were relatively broad and basically belonged to basic cellular BPs, suggesting that 12% SMS inhibited the osteogenic differentiation of PPDLSCs but did not change their mRNA traits (Fig. 2E–J).

### LncRNA00638 was identified as a target lncRNA that is closely associated with the osteogenic differentiation of PPDLSCs under 12% SMS loading

**Fig. 3:**
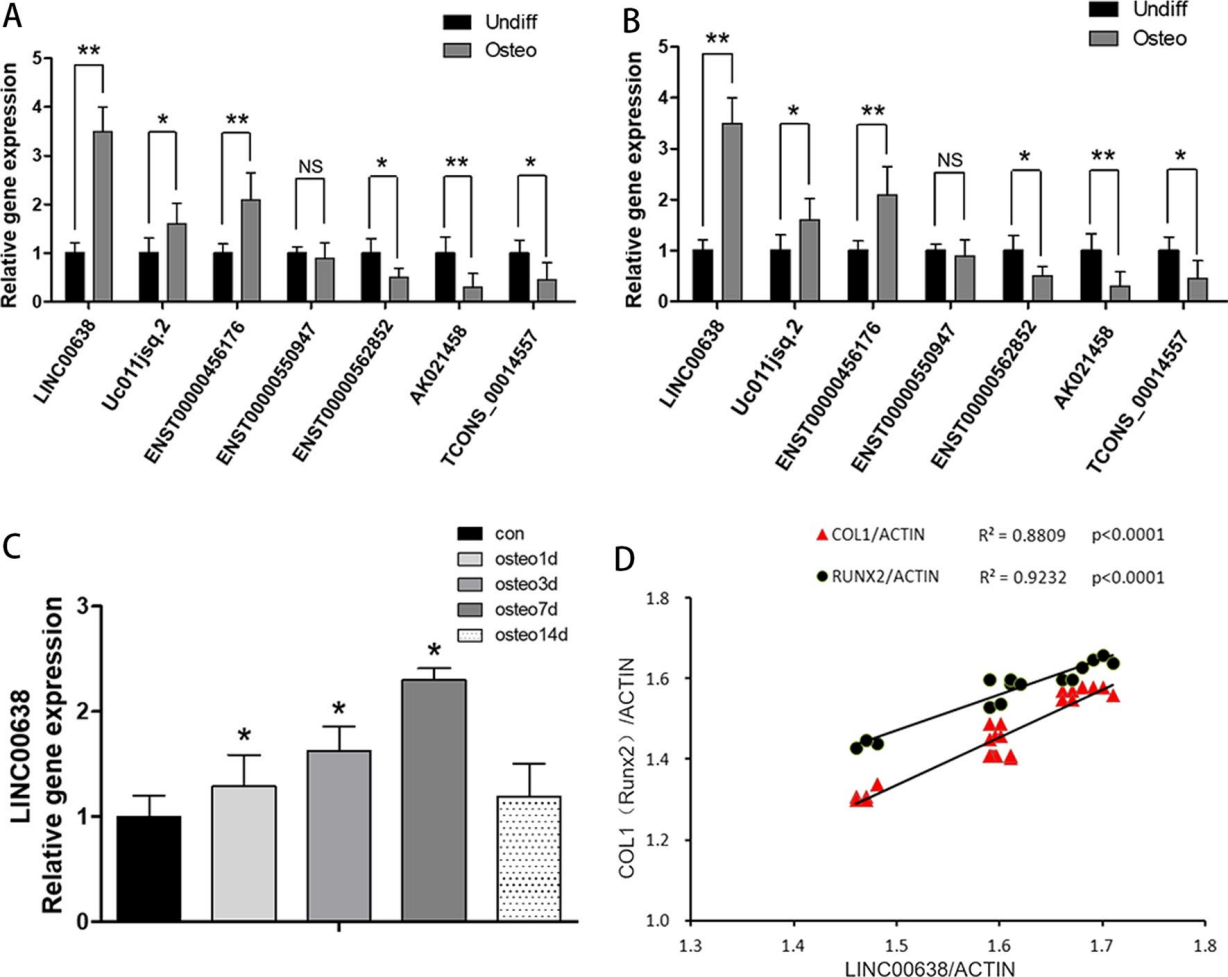
LncRNA00638 is associated with osteogenic differentiation. **A** The microarray results were validated by RT-qPCR. The RT–qPCR and microarray results were consistent for seven of the selected lncRNAs, among which lncRNA00638 showed the most significant difference, while the difference in TCONS_00008000 was not statistically significant. **B** Among the seven lncRNAs for which relative expression was detected by RT-qPCR after 7 d of osteogenic induction, lncRNA00638 expression changed most significantly. **C** Expression levels of lncRNA00638 at 0, 1, 3, 7 and 14 d detected by RT-qPCR. **D** Pearson correlation analysis showed that the expression level of lncRNA00638 was strongly related to Runx2 (*R*^2^ = 0.8935) and Col1 (*R*^2^ = 0.8935) during osteogenic induction. Osteo: osteogenic induction. Undiff: no osteogenic induction. **: *P* < 0.01; *: *P* < 0.05; NS: no significant difference

After that, we screened eight alternative lncRNAs with a fold change > 50 and *p*-value < 0.05, combined with a raw intensity > 100 excluding overlapping sense exon. The RT-qPCR results for seven of the lncRNAs were consistent with the microarray results (Fig. 3A). Then, we assessed whether these lncRNAs could affect the osteogenesis process of PPDLSCs. The RT-qPCR results showed that the expression of lncRNA00638 (ID: ENSG00000258701 in UCSC; ID: NR_024396 in Refseq) changed most significantly after osteogenic induction with 12% SMS (Fig. 3B). In addition, lncRNA00638 expression was continuously elevated with osteogenic induction at 0, 1, 3, 7 and 14 d, peaking at 7 d (Fig. 3C). In addition, the Pearson correlation analysis demonstrated that lncRNA00638 was strongly related to the osteogenic-related genes Runx2 and Col1 during osteogenic induction (Fig. 3D). Therefore, lncRNA00638 was identified as the target lncRNA for subsequent experiments.

### LncRNA00638 promoted the osteogenic differentiation of PPDLSCs under SMS loading

**Fig. 4:**
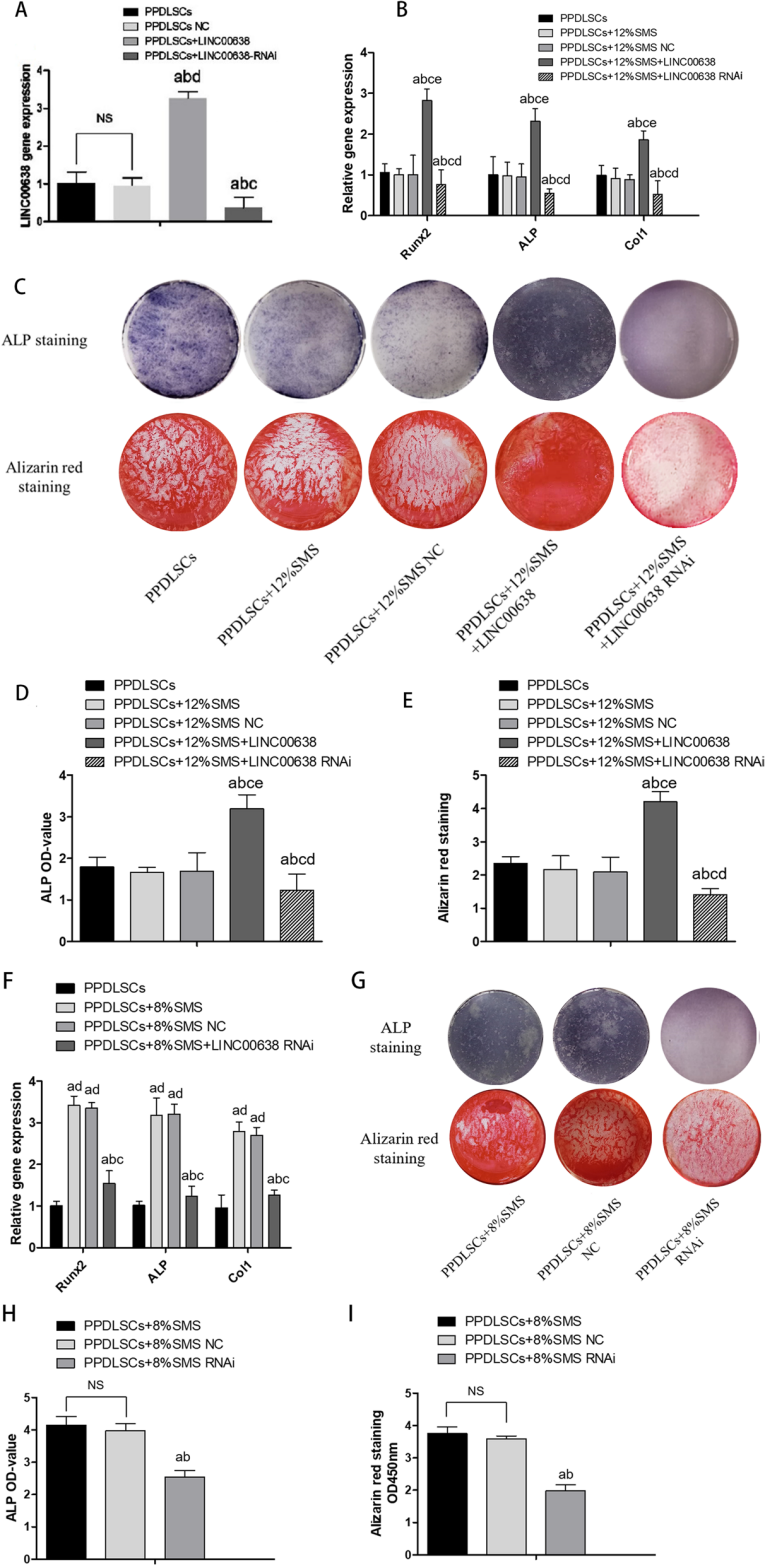
LncRNA00638 promotes osteogenic differentiation.** A** RT-qPCR detection of the infection efficiency of the lncRNA00638-expressing lentivirus. **B** Expression of Runx2, ALP and Col1 was detected by RT-qPCR after 12% SMS loading and 7 days osteogenic induction. **C** Images showing ALP and ARS staining. **D** Quantitative assay of ALP activity. **E** Quantitative assay of ARS staining. **F** Expression of Runx2, ALP and Col1 was detected by RT-qPCR after 8% SMS loading and 7 days of osteogenic induction. **G** Images showing ALP and ARS staining. **H** Quantitative ALP assay results. **I** Quantitative ARS staining results. Data are presented as the mean ± SD a: compared with the PPDLSCs group (*P* < 0.05); b: compared with the PPDLSCs + 12%SMS group (*P* < 0.05); c: compared with the PPDLSCs + 12% SMS NC group (*P* < 0.05); d: compared with the PPDLSCs + 12% SMS lncRNA00638 group (*P* < 0.05); e: compared with the PPDLSCs + 12% SMS lncRNA00638-RNAi (*P* < 0.05)

To explore the biological effect of lncRNA00638 on osteogenic differentiation of PPDLSCs under 12% SMS loading, we constructed lentiviruses for lncRNA00638 upregulation and downregulation and carried out lentiviral transfection (Fig. 4A). The upregulation of lncRNA00638 significantly increased the mRNA levels of the osteogenic genes Runx2, ALP and Col1. Conversely, lncRNA00638 RNA interference (RNAi) significantly decreased the expression levels of these genes (Fig. 4B). The results of ALP assay and ARS staining assay also suggested that lncRNA00638 overexpression increased ALP activity and mineralized bone matrix formation, while lncRNA00638 silencing inhibited osteogenic differentiation (Fig. 4C–E). To further investigate the osteogenic functional role of lncRNA00638 in PPDLSCs osteogenesis under 8% SMS, we silenced lncRNA00638 by also transfecting lncRNA00638-RNAi. The results showed that the expression of Runx2, ALP, and Col1 was decreased in the 8% SMS group (Fig. 4F), and the results of the ALP assay and ARS staining corroborated this trend (Fig. 4G–I). These results suggested that the silencing of lncRNA00638 reduced the osteogenic differentiation of PPDLSCs under 8% SMS, although it is a suitable loading for osteogenesis in an inflammatory environment.

### LncRNA00638 promoted the osteogenic differentiation of PPDLSCs under SMS loading by sponging miR-424-5p to upregulate FGFR1

**Fig. 5:**
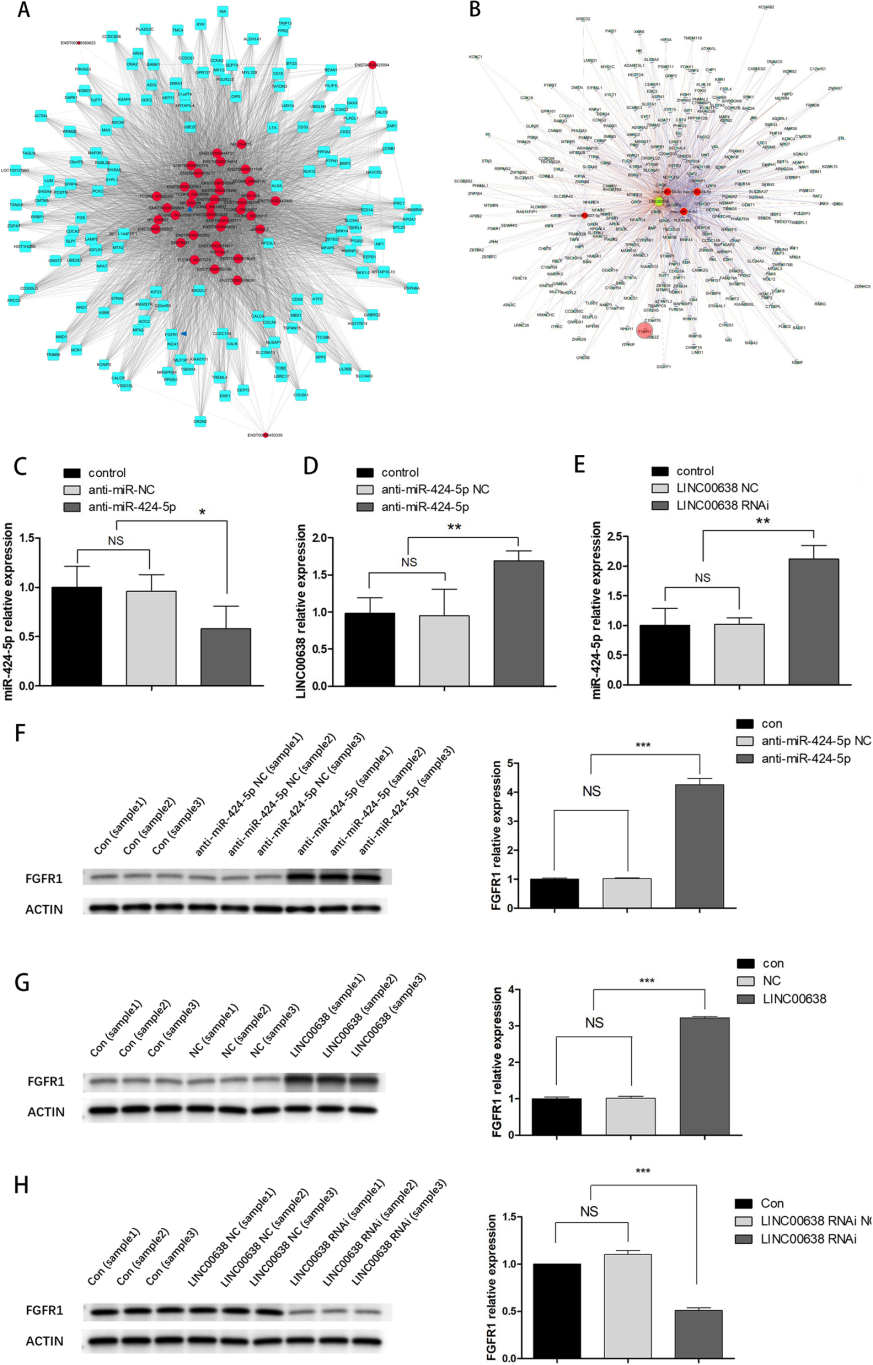
Mechanism by which lncRNA00638 regulates osteogenesis. **A** CNC network mapping for related mRNAs of differentially expressed lncRNAs. **B** CeRNA lncRNA00638 interaction network diagram. Green circle: lncRNA00638; red circle: miR-424-5p; pink circle: FGFR1. **C** Transfection efficiency of anti-miR-424-5p. **D** The expression of miR-424-5p after lncRNA00638-RNAi transfection. **E** The expression of lncRNA00638 after anti-miR-424-5p transfection. **F** Anti-miR-424-5p can promoted FGFR1 expression at the gene and protein levels (Full-length blots are presented in Additional file 2: Fig. S1). **G** FGFR1 gene and protein expression after the overexpression of LncRNA00638 (Full-length blots are presented in Additional file 3: Fig. S2). **H** FGFR1 gene and protein expression after the silencing of lncRNA00638 (Full-length blots are presented in Additional file 4: Fig. S3)

CNC analysis revealed several molecules involved in osteogenic regulatory pathways, such as FGFR1, FPR2, and DAPK1, that might be associated with differentially expressed lncRNAs (Fig. 5A). Through the MiRBase, TargetScan, and UCSC databases, we found that lncRNA00638 contains binding sites for nine miRNAs. Additionally, lncRNA00638 can actively act as a ceRNA for USB1, RPL13, NLRX1, and other genes, suggesting that lncRNA00638 is likely to regulate the target genes by adsorbing microRNA (Additional file 1: Figs. S1 and S2). Then through ceRNA analysis, we found that lncRNA00638 can act as a ceRNA for miR-424-5p and FGFR1 (Fig. 5B). To determine whether lncRNA00638 acts as a sponge that competes with FGFR1 for miR-424-5p binding, we transfected miR-424-5p inhibitor (anti-miR-424-5p) and evaluated its efficacy by RT-qPCR. The results showed that anti-miR-424-5p significantly inhibited the expression of miR-424-5p (Fig. 5C). Meanwhile, the expression of lncRNA00638 was obviously increased (Fig. 5D). In contrast, the expression of miR-424-5p was increased after transfection with lncRNA00638-RNAi (Fig. 5E). The results suggest that miR-424-5p and lncRNA00638 may mutually regulate each other. To explore the relationship between miR-424-5p and FGFR1 expression, we transfected anti-miR-424-5p into PPDLSCs and observed the changes in the gene and protein levels of FGFR1. The results showed that anti-miR-424-5p significantly reduced the mRNA and protein levels of FGFR1 in PPDLSCs (Fig. 5F), which indicated that miR-424-5p could promote the expression of FGFR1 in PPDLSCs. To explore the relationship between lncRNA00638 and FGFR1 expression, we monitored FGFR1 expression after upregulating and downregulating lncRNA00638. FGFR1 expression was significantly increased at the mRNA and protein levels after lncRNA00638 overexpression (Fig. 5G). In contrast, lncRNA00638-RNAi transfection negatively regulated the expression level of FGFR1 (Fig. 5H).

## Discussion

OTM is based on remodelling of the periodontal ligament and alveolar bone under mechanical force. PDLSCs play a vital role in bone resorption on the pressure side and bone regeneration on the tension side during this process [18, 19]. Currently, an increasing number of patients with periodontitis seek orthodontic treatment. Existing evidence shows that combined orthodontic-periodontic treatment can effectively control the levels of inflammatory cytokines and has favourable effects on aesthetic restoration, chewing function and the healing of periodontal tissue [20, 21]. However, the tolerance to the application of excessive orthodontic forces on PDLSCs in the periodontitis microenvironment decreases for several reasons, leading to the destruction of periodontal tissue. The molecular mechanism remains unclear. Accordingly, searching for an effective method to promote the osteogenic capacity of PPDLSCs under SMS is a focus of current research to optimize orthodontic treatment in patients with periodontitis.

In our previous study, different levels of SMS were applied to HPDLSCs and PPDLSCs respectively, and our experiments showed that SMS of 8% was the best for the capability of PPDLSCs, while the best force value for HPDLSCs was 12% SMS [22]. In the present study, we chose these two different levels of SMS and made a lateral comparison between PPDLSCs and HPDLSCs, the results of which are consistent with the previous study indicating that the inflammatory microenvironment attenuates the response of PPDLSCs to mechanical stimuli. Under 12% SMS, the osteogenic capability of HPDLSCs was promoted to a greater extent, while that of PPDLSCs was damaged. However, under conditions of 8% SMS, PPDLSCs showed relatively better osteogenic ability while HPDLSCs did not achieve the best performance. These results fully demonstrate that 12% SMS was the most significant force value.

As a recent research hotspot, lncRNAs play a vital role in many physiological processes and diseases, such as autoimmune, cardiovascular and neurodegenerative diseases. For example, the lncRNA-ANCR was proven to promote osteoblast differentiation by activating the classical Wnt signalling pathway and regulating the expression levels of osteogenic markers such as OCN and Runx2 [8]. Microarray gene chips are one of the important tools to predict and validate the functional roles of lncRNAs. However, existing research represents just the tip of the iceberg. To investigate the mechanisms by which lncRNAs regulate osteogenesis, we performed microarray expression profiling of lncRNAs and screened differentially expressed lncRNAs in PPDLSCs before and after 12% SMS loading. LncRNA00638 was selected as the target lncRNA because it showed the most significant difference in expression by RT-qPCR checking, and the expression levels of lncRNA00638 and the osteogenesis-related genes Runx2/Col1 were highly correlated. To determine the osteogenic capacity of lncRNA00638, we constructed lncRNA00638 overexpression and knockdown lentiviral vectors, and our experiments revealed that lncRNA00638 promoted the osteogenic differentiation of PPDLSCs under 12% SMS. To further investigate the effect of LncRNA00638 on PPDLSCs under appropriate orthodontic force, we silenced the gene after loading 8% SMS loading and found that osteogenesis was also reduced. These results all suggest that lncRNA00638 is a promising target for regulating bone formation in PPDLSCs under mechanical loading conditions.

To further explore the regulatory mechanism of lncRNA00638, bioinformatics analysis and ceRNA network analyses demonstrated that lncRNA00638 may act as a ceRNA with miR-424-5p to regulate FGFR1. There are several molecules involved in osteogenesis regulatory pathways, such as FGFR1, FPR2 and DAPK1. They may be related to differentially expressed lncRNAs, among which FGFR1 plays pivotal roles in bone formation, development, and maturation [23, 24]. Under stimulation from physical signals, the activation of FGFR1/extracellular signal-regulated kinase (FGFR1/ERK) is an important mechanism involved in the regulation of bone formation and remodelling [25–27]. In the present study, the microarray expression profile of PPDLSCs with 12% SMS loading revealed a 4.71-fold change in FGFR1 gene expression, indicating a close connection with osteogenesis. It has been reported that miR-424-5p can mediate the FGF2/FGFR1 signalling pathway, thereby affecting the osteogenic differentiation of marrow stromal cells (MSCs) cells under oxidative stress conditions [28]. Yang et al. reported that overexpression of miR-424-5p can downregulated FGFR1 expression in haemangioma endothelial cells, and inhibition of miR-424-5p upregulated FGFR1 expression, suggesting that miR-424-5p can indirectly interfere with ERK1/2 phosphorylation by inhibiting the FGF2/FGFR1 pathway, thereby affecting cell proliferation [29]. In other words, miR-424-5p can actively participate in the functional regulation of FGFR1. Therefore, we hypothesized that both lncRNA00638 and FGFR1 are targets of miR-424-5p and that lncRNA00638 competes with FGFR1 for binding to miR-424-5p. The following experimental results obtained by RT-qPCR and WB corroborated this hypothesis. First, the expression level of miR-424-5p was found to be negatively correlated with that of lncRNA00638. Second, overexpression of lncRNA00638 promoted the gene and protein expression of FGFR1; silencing lncRNA00638 inhibited the gene and protein expression of FGFR1. Third, silencing the expression of miR-424-5p increased the expression of FGFR1 in PPDLSCs. In summary, lncRNA00638 may act as a ceRNA for miR-424-5p to compete with FGFR1 and thus may participate in the regulation of osteogenic differentiation in PPDLSCs under SMS loading.

Although further evidence of direct binding between these genes is needed, our study demonstrates for the first time that the osteogenic differentiation of PPDLSCs is controlled by the lncRNA00638/miRNA-424-5p/FGFR1 regulatory network under SMS, providing new insights for improving osteogenesis ability and orthodontic efficiency in periodontitis patients.

## Conclusion

In this study, PPDLSCs and HPDLSCs exhibited significantly different cellular responses to 12% SMS. Then differentially expressed lncRNA/mRNA profiles under 12% SMS loading in PPDLSCs were first identified. We confirmed that lncRNA00638 actively participates in the regulation PPDLSC osteogenic differentiation under SMS loading. Then, mechanistic research indicated that lncRNA00638 may act as a sponge competing with FGFR1 for miR-424-5p binding. These results suggest that lncRNAs can regulate the osteogenic ability of PDLSCs under mechanical strain. Nevertheless, comprehensive analyses are still needed to elucidate the details of the relevant molecular mechanisms.

## Supplementary Information

**Additional file 1: Fig. S1** Structure diagram of microRNA with binding site to lncRNA00638. A mi R-16-5p. B mi R-17-3p. C mi R-21-3p. D mi R-106b-5p. E mi R-195-5p. F mi R-211-3p. G mi R-424-5p. H mi R-503-5P. I mi R-3607-5p.** Fig. S2** LncRNA00638 actively participates in the ceRNA process.

**Additional file 2**. Full-length blots of Fig. 5F.

**Additional file 3**. Full-length blots of Fig. 5G.

**Additional file 4**. Full-length blots of Fig. 5H.

## Data Availability

All data generated or analysed during this study are included in this published article (and its Additional file 1). The datasets generated and analysed during the current study are available in OMIX (https://ngdc.cncb.ac.cn/omix) datasets.

## Abbreviations

PDLSCs: Periodontal mesenchymal stem cells
PPDLSCs: Periodontal mesenchymal stem cells obtained from the periodontitis patients
HPDLSCs: Periodontal mesenchymal stem cells obtained from healthy people
SMS: Static mechanical strain
lncRNAs: Long non-coding RNAs
ceRNA: Competing endogenous RNA
FGFR1: Fibroblast growth factor receptor 1
α-MEM: α-Minimum essential medium
FBS: Foetal bovine serum
KEGG: Kyoto encyclopedia of genes and genomes
GO: Gene ontology
BP: Biological process
CC: Cellular component
MF: Molecular function
CNC: Coding-noncoding gene co-expression
ARS: Alizarin red S
ALP: Alkaline phosphatase
RT-qPCR: Real-time quantitative polymerase chain reaction
cDNA: Complementary DNA
MSCs: Marrow stromal cells
