# Supplementary material for: LncRNA00638 promotes the osteogenic differentiation of periodontal mesenchymal stem cells from periodontitis patients under static mechanical strain
Source: Stem Cell Res Ther. 2023 Jul 11;14:177. doi: 10.1186/s13287-023-03404-6 (PMC10337197; doi:10.1186/s13287-023-03404-6)
Supplement: Supplementary file 1 — Additional file 1: Fig. S1 Structure diagram of microRNA with binding site to lncRNA00638. A mi R-16-5p. B mi R-17-3p. C mi R-21-3p. D mi R-106b-5p. E mi R-195-5p. F mi R-211-3p. G mi R-424-5p. H mi R-503-5P. I mi R-3607-5p. Fig. S2 LncRNA00638 actively participates in the ceRNA process. [file 13287_2023_3404_MOESM1_ESM.docx]

Supplemental Fig. 1 Structure diagram of microRNA with binding site to lncRNA00638. A mi R-16-5p. B mi R-17-3p. C mi R-21-3p. D mi R-106b-5p. E mi R-195-5p. F mi R-211-3p. G mi R-424-5p. H mi R-503-5P. I mi R-3607-5p.

Supplemental Fig. 1 LncRNA00638 actively participates in the ceRNA process.
