# Supplementary figures and images for: LncRNA00638 promotes the osteogenic differentiation of periodontal mesenchymal stem cells from periodontitis patients under static mechanical strain
Source: Stem Cell Res Ther. 2023 Jul 11;14:177. doi: 10.1186/s13287-023-03404-6 (PMC10337197; doi:10.1186/s13287-023-03404-6)

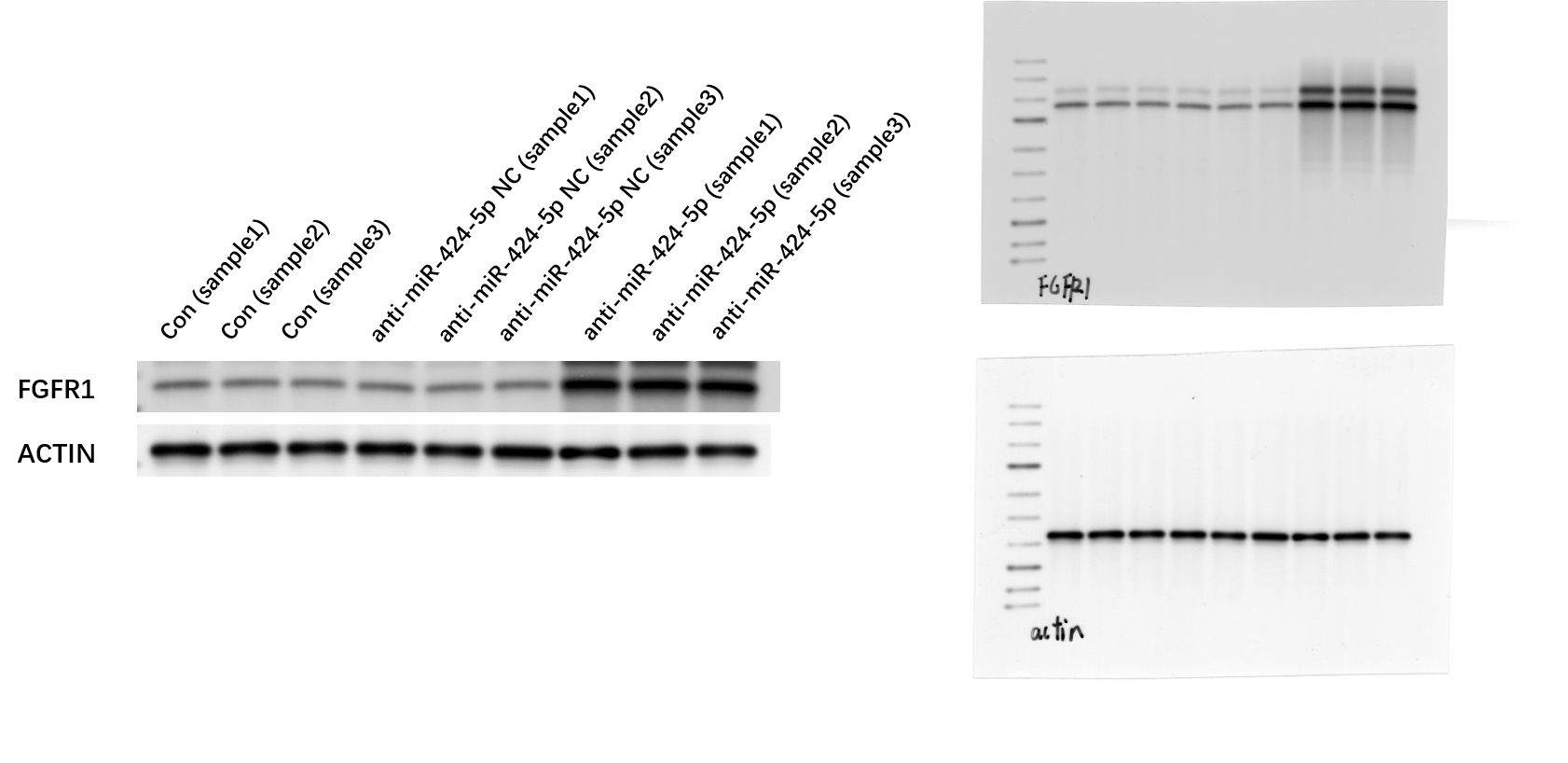

Supplement: Supplementary file 2 — Additional file 2. Full-length blots of Fig. 5F. [file 13287_2023_3404_MOESM2_ESM.tif]

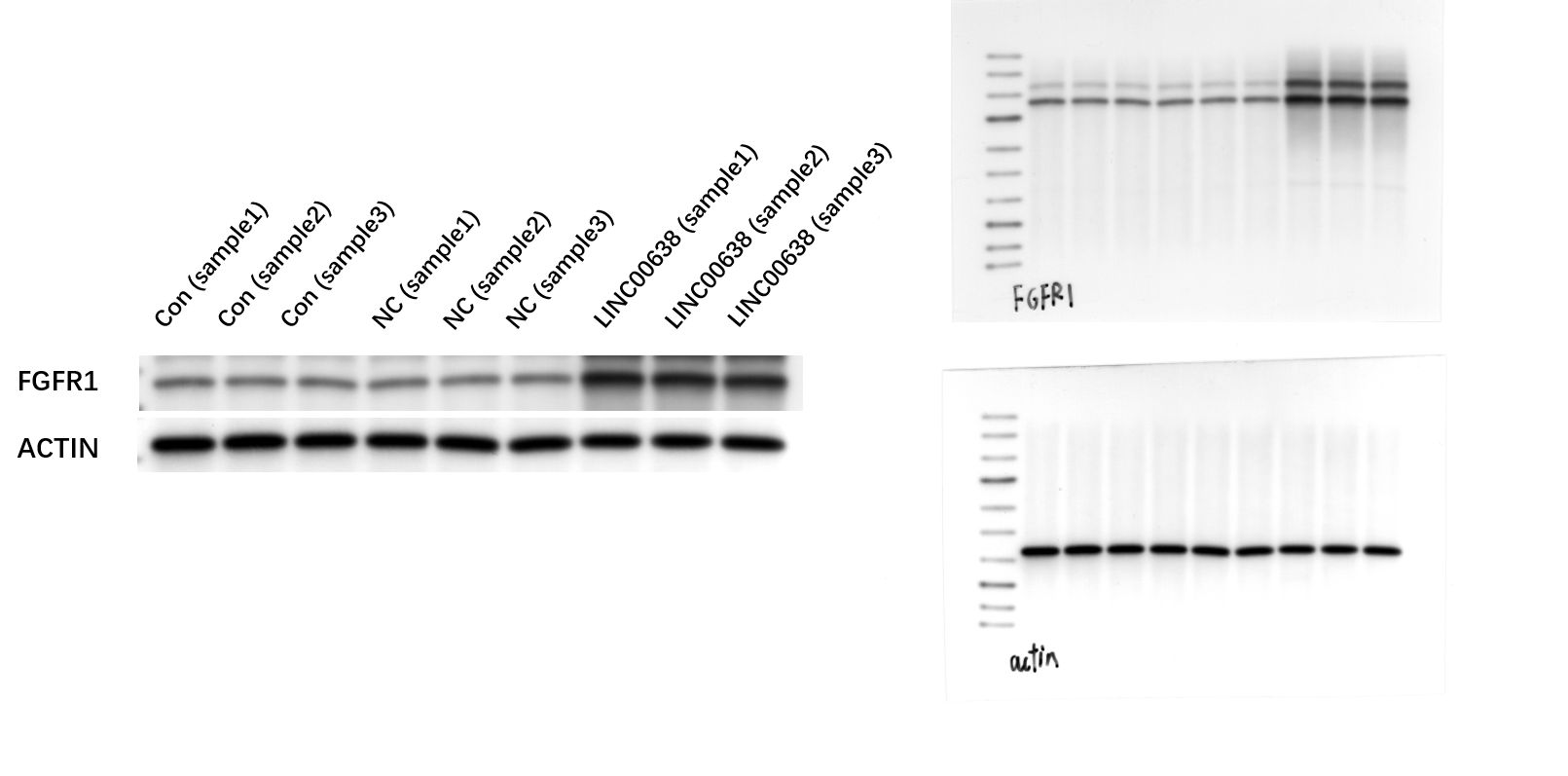

Supplement: Supplementary file 3 — Additional file 3. Full-length blots of Fig. 5G. [file 13287_2023_3404_MOESM3_ESM.tif]

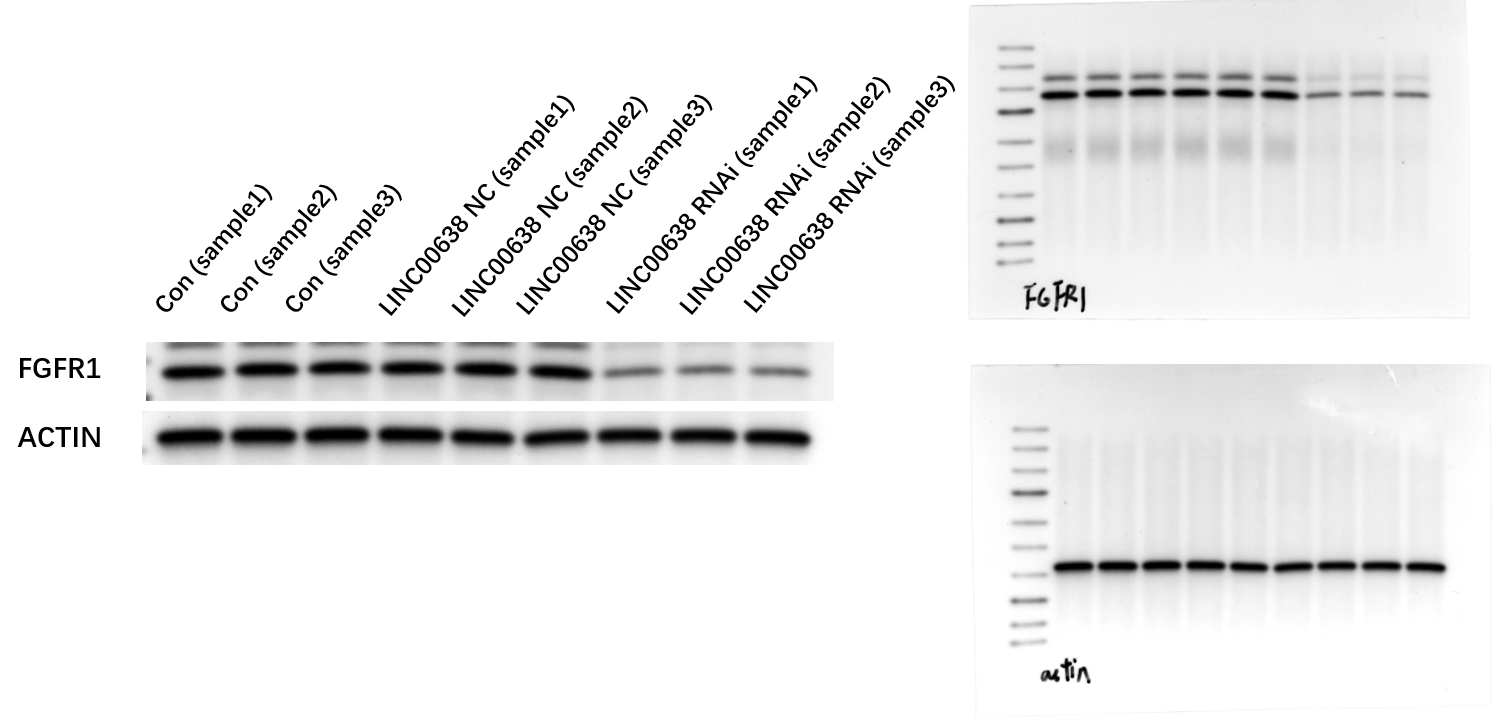

Supplement: Supplementary file 4 — Additional file 4. Full-length blots of Fig. 5H. [file 13287_2023_3404_MOESM4_ESM.tif]
